# *In utero* and lactational exposure to tenofovir disoproxil fumarate and bone health outcomes: a scoping review of 31 studies

**DOI:** 10.3389/fendo.2026.1798685

**Published:** 2026-09-03

**Authors:** Xia Ji, Yuanzhong Wang, Zefan Zhang, Kok-Lun Pang, Xiong Khee Cheong, Norliza Muhammad, Kok-Yong Chin

**Affiliations:** 1 Department of Pharmacology, Faculty of Medicine, Universiti Kebangsaan Malaysia, Cheras, Malaysia; 2 Department of Gynaecology and Obstetrics, Inner Mongolia Baogang Hospital, Baotou, China; 3 Department of Obstetrics & Gynaecology, Tianjin Medical University General Hospital, Heping, China; 4 Chongqing Chemical Industry Vocational College, Changshou, China; 5 Department of Ultrasound Medicine, Inner Mongolia Baogang Hospital, Baotou, China; 6 Jeffrey Cheah School of Medicine and Health Sciences, Monash University Malaysia, Bandar, Sunway, Malaysia; 7 Infectious Disease Unit, Department of Internal Medicine, Faculty of Medicine, Universiti Kebangsaan Malaysia, Cheras, Malaysia

**Keywords:** HBV, highly active antiretroviral therapy, HIV, osteoporosis, pregnancy

## Abstract

**Introduction:**

Tenofovir disoproxil fumarate (TDF) is a key antiretroviral agent for preventing mother-to-child transmission of human immunodeficiency virus (HIV) and treating chronic hepatitis B (HBV) during pregnancy. However, concerns persist regarding its potential effects on bone metabolism during foetal development and postpartum recovery.

**Objective:**

This scoping review synthesised evidence on the impact of *in utero* and lactational TDF exposure on bone health in offspring and mothers.

**Methods:**

A systematic search of PubMed, Scopus, Web of Science and the China National Knowledge Infrastructure identified randomised controlled trials, cohort studies, and observational studies assessing bone mineral density, bone mineral content, growth, or bone turnover markers in relation to perinatal TDF exposure. Thirty-one studies met the inclusion criteria.

**Results:**

Most studies reported no clinically significant long-term effects of TDF on paediatric bone mineralisation, growth, or metabolism from infancy to mid-childhood. A transient reduction in neonatal bone mineral content was noted in a few studies, but its clinical relevance appeared limited. In contrast, TDF use in mothers was consistently associated with accentuated bone loss during pregnancy and lactation, particularly at the hip, with incomplete recovery postpartum, indicating potential long-term skeletal risk.

**Conclusions:**

*In utero* and lactational exposure to TDF had minimal impact on paediatric bone development but was consistently linked to maternal bone loss and incomplete skeletal recovery. While the benefits of TDF in preventing vertical transmission of HIV and HBV outweigh these risks, the findings highlight the importance of ongoing monitoring of maternal bone health.

## 1 Introduction

Tenofovir disoproxil fumarate (TDF) has been a cornerstone antiretroviral agent for over two decades, owing to its potent antiviral efficacy and favourable resistance profile (1). It has been widely used for the treatment and prevention of Human Immunodeficiency Virus (HIV) infection and served as a first-line therapy for chronic hepatitis B virus (HBV) infection (2, 3). A critical application of TDF lies in the prevention of mother-to-child transmission (PMTCT) of these viruses, making its safety during pregnancy and lactation a matter of considerable clinical importance (4, 5). Despite its established therapeutic value, TDF has been consistently associated with bone toxicity in non-pregnant adults (6). It was shown to cause modest yet significant reductions in bone mineral density (BMD) and elevations in bone turnover markers, partly due to proximal renal tubular dysfunction affecting mineral homeostasis (7; J. 8). These skeletal effects raised legitimate concerns about potential risks during periods of heightened vulnerability, such as foetal skeletal development and the maternal postpartum phase.

Pregnancy and lactation were characterised by dynamic alterations in calcium and bone metabolism to support foetal mineralisation and milk production (9). During these periods, physiological bone resorption increased, rendering the maternal skeleton more susceptible to additional stressors. The concurrent use of a bone-active drug such as TDF might therefore have compounded these effects (10). Existing findings on TDF exposure during pregnancy had been mixed. While several studies reported no adverse effects on infant growth or bone development (11–13), others observed transient reductions in neonatal bone mineral content (BMC) or alterations in biochemical markers of bone metabolism (7, 14). For mothers, longitudinal studies suggested that TDF use during pregnancy and lactation accentuated bone loss and delayed skeletal recovery (15, 16).

More recent clinical trials provided both reassurance and caution. A randomised controlled trial in China showed that infants exposed to TDF *in utero* for HBV prevention exhibited normal growth, bone mineralisation, and neurodevelopment at 192 weeks of age (17). Similarly, TDF-based pre-exposure prophylaxis (PrEP) during pregnancy did not affect infant BMC at 6–74 weeks (18, 19). However, other studies, including those in older adults with chronic HBV, revealed an increased fracture risk associated with prolonged TDF use (20). These divergent findings underscore the complexity of interpreting TDF’s skeletal effects across populations and life stages.

Given the variability in exposure timing, population type, and bone health assessment methods, consensus on the impact of TDF on maternal and infant bone health had remained elusive. Therefore, this scoping review aimed to systematically map and synthesise existing evidence on the effects of *in utero* and lactational exposure to TDF. Its objectives were: (1) to summarise findings on foetal, neonatal bone mineralisation and growth following TDF exposure; and (2) to elucidate maternal bone changes associated with TDF use during pregnancy and lactation. By consolidating available data, this review sought to clarify the risk-benefit profile of TDF in the perinatal period and inform safer clinical practice.

## 2 Materials and methods

This scoping review was conducted following the methodological framework outlined by Arksey and O’Malley (21) and the Joanna Briggs Institute (JBI) methodology for scoping reviews. and was reported in accordance with the Preferred Reporting Items for Systematic Reviews and Meta-Analyses Extension for Scoping Reviews guidelines (PRISMA-ScR) (22). The protocol of this scoping review has been registered in the Open Science Framework (OSF) (Url: https://osf.io/9dexj).

### 2.1 Identifying the research question

The review sought to address the following question: What are the effects of TDF on bone health during pregnancy? Specifically, we aimed to map existing evidence on maternal TDF exposure and its impact on skeletal outcomes in mothers and their offspring, including BMD, BMC, fracture risk, and bone remodelling.

### 2.2 Identifying the relevant studies

A systematic search was carried out in June 2026 across PubMed, Web of Science, Scopus, and the China National Knowledge Infrastructure (CNKI). PubMed, Web of Science and Scopus were selected because they are the most comprehensive biomedical and multidisciplinary databases, respectively. CNKI was included to capture Chinese-language literature, given the substantial body of research on TDF use in pregnancy conducted in China. The search terms included combinations of: (“tenofovir disoproxil fumarate” OR “TDF”) AND (“osteoporosis” OR “bone” OR “skeleton” OR “osteoblast*” OR “osteoclast*” OR “osteocyte” OR “fracture*” OR “bone mineral density” OR “bone mineral content” OR “vitamin D” OR “calcium” OR “bone turnover” OR “alkaline phosphatase” OR “BAP” OR “C-terminal telopeptide of type I collagen” OR “CTX” OR “parathyroid hormone” OR “PTH”) AND (“pregnan*” OR “gestation” OR “antenatal”). Reference lists of relevant articles were also screened to identify additional studies.

### 2.3 Eligibility criteria

Studies were eligible if they were full-text reports containing original data on maternal TDF exposure during pregnancy, evaluated bone-related outcomes in mothers or their infants, and were published in English or Mandarin. Human studies with experimental design (clinical trial) and observational designs were eligible. Articles were excluded if they were reviews, commentaries, editorials, case reports, or if they did not report skeletal outcomes.

### 2.4 Study selection criteria

All retrieved records were imported into EndNote (version 21.4, Clarivate, London, UK) for deduplication (23). Two authors (XJ and YZW) independently screened titles and abstracts, followed by full-text assessment of potentially eligible articles. Disagreements were resolved by discussion or by consultation with a third reviewer (KYC). All authors involved in screening are fluent in both English and Mandarin.

### 2.5 Data extraction

Data were extracted independently by two authors (XJ and YZW) using a standardised form. Extracted information included study design, population, sample size, details of TDF exposure (dose, duration, timing), comparator groups, bone-related outcomes, and key findings. The data were tabulated, and inconsistencies were resolved by consensus among the authors.

### 2.6 Data synthesis

The data were synthesised qualitatively to cover the wide range of bone outcomes reported. The data were presented according to the infants’ developmental stages and separately for the mother. Consistent with the objectives of a scoping review, which is to map the existing evidence rather than to critically appraise study quality or synthesise effect estimates, a formal risk of bias assessment of the included studies was not performed.

## 3 Results

### 3.1 Study selection

**Figure 1:**
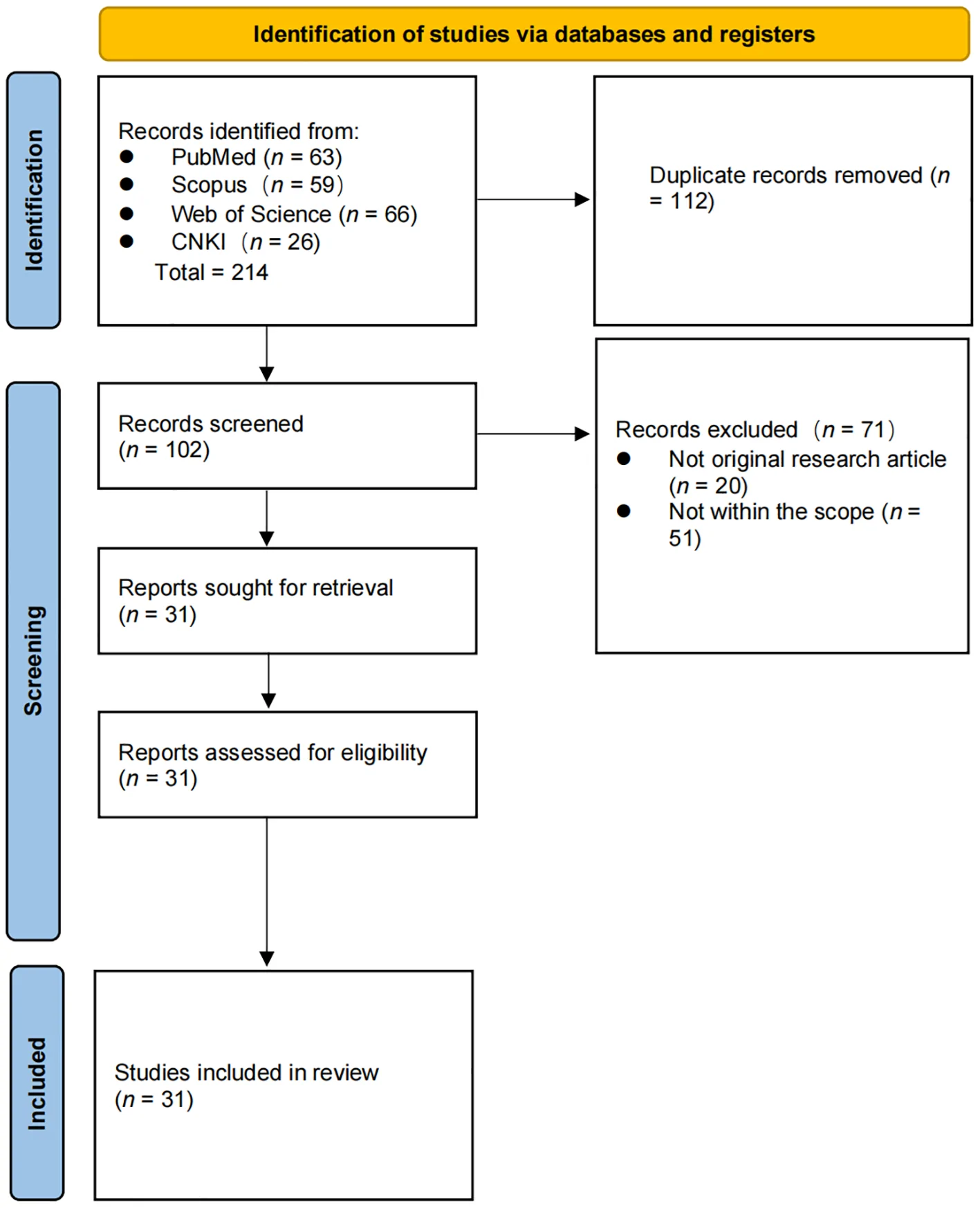
Flowchart showing selection of articles.

The initial search retrieved 214 records. After removing duplicates (n = 112), 102 unique studies remained. Following title and abstract screening, 71 were excluded for the following reasons: not original research (n = 20), outside the scope of this review (n = 51). A total of 31 studies met the final inclusion criteria. The study selection process is illustrated in **Figure 1**.

### 3.2 Study characteristics

The 31 included studies comprised over 6,000 participant entries as reported in the individual studies, though some originated from overlapping parent cohorts and may therefore include the same individuals. These studies were grouped according to their primary focus on either offspring or maternal bone outcomes. Most studies employed observational designs (prospective or retrospective cohorts), while a minority were randomised controlled trials or secondary analyses.

Twenty studies focused exclusively on infant and child outcomes (7, 14, 15, 17–19, 24–37). These investigations primarily evaluated bone mineralisation, growth, and bone metabolism in HIV-exposed uninfected infants and children, or in infants born to mothers with HBV. The primary exposure assessed was *in utero* TDF exposure, with several studies also examining postnatal exposure through breastfeeding (24, 34, 38, 39). Key comparison groups included infants unexposed to TDF but exposed to other antiretrovirals [e.g., zidovudine or azidothymidine (AZT)], as well as infants with no antiretroviral exposure. The primary outcomes measured across these studies were BMC and BMD assessed by dual-energy X-ray absorptiometry (DXA), quantitative ultrasound parameters, anthropometric growth Z-scores (e.g., weight-for-age, height-for-age, head circumference), and serum or urinary biomarkers of bone turnover such as bone-specific alkaline phosphatase (BAP) and C-terminal telopeptide of type I collagen (CTX). Findings on infant bone mineralisation were mixed, with most studies reporting no significant adverse effects (14, 15, 17–19, 24–28, 31, 32, 35–37), although lower neonatal or infant BMC was reported in some (33, 34).

Eleven studies focused on maternal bone outcomes during the postpartum period (16, 38–47). These studies enrolled women with HIV (WWH) or women using TDF/emtricitabine (FTC) as PrEP. The exposures investigated consisted of maternal TDF-based antiretroviral therapy (ART) initiated during pregnancy or postpartum. Comparison groups included HIV-negative women, women with HIV receiving non-TDF regimens, or women who deferred TDF-based PrEP until after delivery. Maternal outcomes were primarily assessed through DXA measurements of BMD at the lumbar spine, total hip, and femoral neck, as well as biochemical markers of bone turnover and mineral metabolism, including parathyroid hormone (PTH), calcium, and phosphate. Several studies reported greater bone loss in mothers receiving TDF-based ART during lactation, with some indicating incomplete post-lactation recovery at specific skeletal sites (43; Nabwire et al., 2023; 39, 45).

The study populations were geographically diverse, with substantial representation from sub-Saharan Africa (e.g., Uganda, Malawi, South Africa) (7, 16, 24, 25, 38, 41–44 39, 45), Asia (e.g., China, Thailand) (28, 31, 32, 36, 37, 46, 47), and multi-country international cohorts (14, 15, 19, 26, 27, 34, 35, 40). An overview of all the studies included is presented in **Table 1**.

**Table 1:** Summary of studies on TDF and bone health, categorised by population and outcome focus.

Subcategory	Outcome domain	No. of studies (n)	Exposure & comparators	Key assessments/markers	References
Maternal bone outcomes	BMD	9	TDF-based ART or PrEP vs. non-TDF regimens (DTG, EFV-based, or placebo)	DXA/X-ray (lumbar spine, hip, femoral neck)	(15, 18, 30, 39, 41–43, 45, 46)
	Bone metabolism & biomarkers	2	Maternal TDF-based ART vs. non-TDF regimens	P1NP, CTX, PTH, Ca, P	(16, 44)
	Renal & indirect bone safety	2	TDF therapy during pregnancy vs. controls	Creatinine clearance, serum ALP	(18, 47)
Offspring bone outcomes	BMC/BMD	10	*In utero*/lactational TDF exposure vs. other ARVs or unexposed	DXA (infant/child BMC, BMD)	(7, 26, 28, 29, 31–34, 36, 37)
	Growth & anthropometrics	6	*In utero*/lactational TDF exposure vs. controls	Z-scores (weight, height, HC)	(17, 25, 27, 35, 38, 40)
	Bone metabolism & biomarkers	5	*In utero*/lactational TDF exposure vs. controls	BAP, CTX, Ca, P, PTH	(14, 24, 35, 38, 40)
	Foetal bone development	1	Duration of *in utero* TDF exposure	Long bone length (femur, humerus) by ultrasound	(27)
	Long-term outcomes (>1 year)	4	*In utero* TDF exposure vs. controls	Growth, BMD, bone markers in childhood	(17, 25, 32, 37)

ALP, alkaline phosphatase; ART, antiretroviral therapy; ARVs, antiretroviral drugs; BAP, bone-specific alkaline phosphatase; BMC, bone mineral content; BMD, bone mineral density; Ca, calcium; CTX, C-terminal telopeptide of type I collagen; DTG, dolutegravir; DXA, dual-energy X-ray absorptiometry; EFV, efavirenz; FTC, emtricitabine; HC, head circumference; P, phosphate; P1NP, N-terminal propeptide of type I procollagen; PrEP, pre-exposure prophylaxis; PTH, parathyroid hormone; TDF, tenofovir disoproxil fumarate.

### 3.3 Foetal and neonatal bone mineralisation and growth

Evidence from multiple studies suggested that *in utero* exposure to TDF did not adversely affect foetal or neonatal growth trajectories or bone mineralisation in most settings. An observational study of pregnant women with HBV infection further supported the safety profile of TDF, reporting no significant differences in neonatal birth weight, length, or head circumference among infants exposed to full-term or late-pregnancy maternal TDF therapy compared with unexposed infants (47). A multicentre observational cohort found no significant differences in growth parameters, tibial speed of sound, or bone turnover markers between TDF-exposed and unexposed HIV-uninfected children, although PTH levels were slightly lower but remained within the reference range (35). Similarly, a large study in South Africa reported no association between the duration of maternal TDF exposure and foetal femur or humerus growth, and birth length Z-scores were unaffected (27).

Randomised trial evidence also supported these findings. In the CAP016 trial, infants exposed *in utero* to maternal TDF/FTC-based PrEP showed comparable BMC at multiple time points up to 18 months of age, even among mothers with high adherence (19). Analysis of meconium samples confirmed detectable TDF in nearly all exposed infants, yet drug concentrations did not correlate with neonatal growth or BMC outcomes (26).

However, not all findings were consistent. A study of renal and bone biomarkers in HIV-exposed uninfected neonates suggested that third-trimester TDF exposure was associated with increased urinary phosphate loss and lower vitamin D levels, although no significant effect on markers of bone resorption was observed, indicating a limited short-term impact on bone turnover (14). One study reported significantly lower neonatal whole-body BMC (−12%) among TDF-exposed infants compared with unexposed peers, an effect that persisted after adjustment for maternal and infant covariates (33). By contrast, a smaller pilot study in China found lower mean BMC and BMD in exposed infants at birth and six months, but these differences were not statistically significant after adjustment (28).

Additional evidence was provided by a secondary analysis of bone radiographic data from the HIV Prevention Trials Network (HPTN) 057 pharmacokinetic trial, which evaluated peripartum TDF exposure. Hand, wrist, and spine radiographs were obtained from 89 HIV-exposed infants at 3 days and 12 weeks of age across three TDF dosing cohorts in Malawi and Brazil. Metaphyseal lucency was identified in one infant in Brazil and two infants in Malawi. Bone age discrepancies were observed in 15% of Brazilian infants and 9% of Malawian infants. Additional abnormalities identified in the Malawian cohort included seven cases of wrist osteopenia, two cases of spinal osteopenia, and three other skeletal abnormalities. The prevalence of abnormal radiographic findings increased from 9.2% on day 3 to 17.2% on week 12. Nevertheless, because TDF exposure before birth had been minimal, these abnormalities were considered unlikely to be attributable to TDF. Instead, untreated maternal HIV infection and poor maternal nutritional status were proposed as more plausible contributors to impaired foetal skeletal development, warranting further investigation in prospective cohort studies (30).

More recent evidence from the prospective Gumba cohort in Uganda further extended these findings. HIV-exposed uninfected infants with *in utero* and breastfeeding exposure to maternal TDF-containing ART exhibited slower postnatal growth and lower whole-body BMC and bone area during early infancy than HIV-unexposed controls. However, areal BMD remained comparable after adjustment for body size, and no differences were observed in biochemical markers of bone formation. In contrast, bone resorption marker concentrations were significantly higher among HIV-exposed infants, suggesting altered bone remodelling despite preserved bone mineralisation relative to body size. These findings indicated that early growth deficits and changes in bone metabolism might occur in HIV-exposed uninfected infants, although the relative contributions of maternal HIV infection, TDF exposure, and other maternal factors could not be distinguished and require further investigation (29).

Taken together, the available evidence suggested that *in utero* exposure to TDF was generally not associated with impaired foetal or neonatal bone health, although some studies reported reduced neonatal BMC. These findings are summarised in **Table 2**, which provides an overview of study populations, interventions, exposure duration, and key outcomes.

**Table 2:** Summary of studies evaluating *in utero* exposure to TDF and bone health outcomes in infants.

Study (year)	Population/model	Intervention	Exposure/duration	Key findings	Conclusion
Viganò et al., 2011 (35)	HIV-uninfected children born to HIV infected mothers (n=68)	HAART with TDF (n=33) vs HAART without TDF (n=35)	Median gestational exposure: 25 vs 34 weeks	Growth trajectories normal; tibial SOS, BAP, CTX similar; PTH slightly lower but within range; urinary calcium slightly higher	TDF exposure did not impair growth, bone mineralisation, or metabolism in early childhood
Jao et al., 2016 (27)	646 HIV-infected pregnant women/foetus dyads, South Africa	Maternal TDF containing cART	2–40 weeks *in utero*	No association between TDF duration and femur/humerus length z-scores; birth length unaffected	TDF exposure was not associated with impaired fetal long bone growth
Reddy et al., 2024 (19)	HIV unexposed infants, South Africa (n=335)	Maternal TDF/FTC PrEP	2nd trimester to delivery (median 19 weeks)	Similar whole-body and lumbar spine BMC between immediate vs deferred PrEP groups up to 18 months, including high-adherence subgroup	TDF/FTC PrEP did not impair bone mineralisation in early life
Himes et al., 2020 (26)	HIV exposed, uninfected infants (n=58)	Maternal TDF	≥8 weeks in 3rd trimester; meconium TFV measured	TFV detectable in 55/58; concentrations unrelated to exposure duration/timing; no association with growth or BMC	No concentration-dependent effect; bone outcomes unaffected
Siberry et al., 2015 (33)	Singleton infants ≥36 weeks (TDF-exposed n=74, unexposed n=69)	Maternal TDF	≥8 weeks in 3rd trimester	Exposed infants had 12% lower mean whole-body BMC; adjusted difference -5.3 g (95% CI −9.5 to −1.2); effect persisted after covariate adjustment	Maternal TDF use was associated with significantly lower neonatal BMC; long-term implications require study
Osorio et al. (2017) (30)	89 HIV-exposed infants (56 Malawi, 33 Brazil); hand/wrist & spine X-rays at day 3 and week 12	Cohort 1: maternal 600 mg TDF during labour (no infant dose); Cohort 2: infant 4 mg/kg on days 0,3,5 (no maternal dose); Cohort 3: maternal 900 mg + infant 6 mg/kg on days 0,3,5	Peripartum TDF (maternal intrapartum and/or up to 3 neonatal doses in the first week)	Metaphyseal lucency: Brazil 1 (3%), Malawi 2 (3.6%); wrist osteopenia: Malawi 7 (12.5%) only; spine osteopenia: Malawi 2 (3.6%) only; bone-age anomalies: Brazil 15% vs Malawi 9%; total abnormal films rose from 9.2% (day 3) to 17.2% (week 12); 79% normal at both visits.	TDF unlikely causative; abnormalities attributed primarily to maternal HIV disease severity and/or nutritional deficits; further long-term studies recommended.
Kourtis et al., 2018 (28)	Infants of HIV/HBV-coinfected mothers, China (n=27)	Maternal TDF-containing cART	*In utero*, assessed at birth and 6 months	Exposed infants had lower mean BMC and BMD, but the differences were not statistically significant after adjustment	TDF exposure did not significantly impair infant bone health in the first 6 months
Xiong et al., 2022 (47)	120 pregnant women with HBV infection in China	Maternal TDF therapy (full term or late pregnancy) vs no antiviral treatment	300 mg oral TDF daily; full term or from 24–28 weeks to delivery	No significant differences in maternal renal function or neonatal growth parameters; rare adverse events	Maternal TDF exposure during pregnancy did not adversely affect renal function or fetal growth, supporting overall maternal and neonatal safety
Purswani et al., 2024 (14)	141 HIV exposed uninfected neonates (77 TDF-exposed, 64 unexposed)	Maternal TDF use ≥ 8 weeks in the third trimester	Neonatal samples collected 4–30 days after birth	PTRP declined faster in TDF-exposed neonates; serum 25(OH)D was lower; no significant differences in creatinine, phosphate, eGFR, PTH, or urine NTx/cr	Third-trimester TDF exposure was associated with increased neonatal phosphate excretion and lower vitamin D, but bone resorption markers were unaffected, suggesting limited short-term effect on neonatal bone
Nabwire et al. (2026) (29)	165 Ugandan infants (85 CHEU, 80 CHUU); prospective cohort; anthropometry to 78 weeks; DXA (WB, LS) at 2, 14, 26 weeks; biochemical markers at 14, 26, 52, 78 weeks	Observational: WLH initiated TDF/3TC/EFV during pregnancy (CHEU); HIV-negative mothers (CHUU)	*In utero* + lactational TDF exposure (cumulative; maternal ART initiated during pregnancy, breastfeeding to ~12 month)	Similar size at 2 weeks; CHEU slower growth by 14 weeks; lower weight and fat mass (~0.5 kg) at 26 weeks; stunting at 78 weeks: CHEU 59% vs CHUU 33% (p=0.003); lower WB BMC and BA at 14 & 26 weeks (p=0.03, p=0.0003); no group difference in size-adjusted a BMD; CTX higher in CHEU at 14 weeks (+50.9%, p<0.001) and 78 weeks (+45.6%, p<0.05); no group differences in IGF1, P1NP or OC.	Early growth and bone deficits observed in CHEU by 3 months; altered bone metabolism (higher resorption) may be linked to maternal TDF-ART and/or maternal bone changes; long-term implications require further study.

BAP, bone alkaline phosphatase; BMC, bone mineral content; BMD, bone mineral density; cART, combination antiretroviral therapy; CI, confidence interval; CTX, C-terminal telopeptide of type I collagen; eGFR, estimated glomerular filtration rate; FTC, emtricitabine; HAART, highly active antiretroviral therapy; HBV, hepatitis B virus; HIV, human immunodeficiency virus; NTx/Cr, N-terminal telopeptide of type I collagen to creatinine ratio; PrEP, pre-exposure prophylaxis; PTH, parathyroid hormone; PTRP, photothermal radiometry and modulated luminescence; SOS, (quantitative ultrasound parameter); TDF, tenofovir disoproxil fumarate; TFV, tenofovir.

### 3.4 Infant and early childhood bone health and metabolism

Long-term follow-up studies generally indicated that *in utero* exposure to TDF did not have an adverse effect on infant growth, bone health, or development. In the Development of Anti-Retroviral Therapy in Africa trial, which followed infants of HIV-infected mothers initiating ART during pregnancy for a median of 5.1 years, no significant differences in growth trajectories, adverse renal events, or clinical outcomes were observed between infants with and without tenofovir exposure. Height-for-age Z-scores were slightly lower than the World Health Organisation’s reference, but did not differ by tenofovir exposure, and no bone fractures were reported. Overall, *in utero* tenofovir exposure was not associated with impaired bone health or increased risk of skeletal abnormalities in early childhood (25). In children born to mothers with chronic hepatitis B, no significant differences were observed in congenital malformations, growth, neurodevelopment, or BMD up to 192 weeks of age (17). Similar findings were reported in a cohort of children followed to a median age of four years, in whom growth, renal function, bone markers, and BMD did not differ between TDF-exposed and unexposed groups (37). Comparative data from mothers treated throughout pregnancy further showed that TDF offered superior antiviral efficacy over telbivudine, with no adverse consequences for children’s growth or bone development at five years of age (32).

A randomised, double-blind, placebo-controlled trial from Thailand provided further supportive evidence, reporting no significant differences in infant lumbar spine BMD at 12 months of age between those exposed to maternal TDF until 2 months postpartum and the placebo group (31). Studies conducted in Malawi and Vietnam also supported these conclusions. Infants exposed to TDF *in utero* and during breastfeeding demonstrated normal bone turnover markers, growth, neurodevelopment, and renal function, with only occasional transient or mild biochemical alterations (24, 38, 40). Similarly, a study evaluating TDF for the prevention of HBV mother-to-child transmission reported no adverse effects of breastfeeding on infant BMD among TDF-exposed infants up to 12 months of age, while confirming the regimen’s high efficacy in preventing vertical transmission (36). A sub-study of the International Maternal Paediatric Adolescent Acquired Immunodeficiency Syndrome (AIDS) Clinical Trials Network, Promoting Maternal and Infant Survival Everywhere (PROMISE) trial reported a modest reduction in lumbar spine at 26 weeks among breastfed infants born to TDF-treated mothers; however, the difference was small and deemed unlikely to be clinically significant (34).

Overall, the evidence suggested that maternal TDF use during pregnancy and lactation was not associated with clinically relevant impairment of infant or early childhood bone health. However, isolated reports of reduced bone mineral accrual highlighted the importance of ongoing surveillance. These studies are summarised in **Table 3**, which provides an overview of infant and early childhood outcomes following TDF exposure.

**Table 3:** Summary of studies evaluating infant and early childhood outcomes following *in utero* or lactational exposure to TDF.

Study (year)	Population/model	Intervention	Exposure/duration	Key findings	Conclusion
Pan et al., 2022 (17)	180 infants born to mothers with chronic HBV, China	Maternal TDF vs no treatment during the third trimester	8.6 weeks of fetal exposure	No differences in congenital malformations, BMD, growth, or neurodevelopment; adverse events similar	Foetal TDF exposure was not associated with impaired bone development, growth, or neurodevelopment up to 192 weeks
Wen et al., 2020 (37)	128 children aged 2–7 years, born to HBV-infected mothers	Maternal TDF 300 mg/day from 30–32 weeks of gestation until 1 month postpartum	Follow-up annually; DXA scans for BMD	No differences in growth, renal function, bone markers, or BMD at lumbar spine/hip	Fetal TDF exposure during late pregnancy did not adversely affect long-term bone development, growth, or renal function
Shang et al., 2021 (32)	165 women with chronic HBV and their children (74 TDF, 91 LDT)	Maternal TDF or LDT therapy	Throughout pregnancy, children followed up to 5 years	TDF showed superior antiviral efficacy; no maternal renal/obstetric adverse events; children’s growth and BMD were comparable	Maternal TDF therapy throughout pregnancy did not adversely affect long-term bone development
Floridia et al., 2017 (24)	136 Malawian infants exposed *in utero* and via breastfeeding	Maternal TDF/lamivudine/efavirenz during pregnancy and breastfeeding	*In utero* + 12 months breastfeeding	BAP often above reference; CTX normal; no differences by sex or exposure; maternal ART duration correlated with infant height	Postnatal TDF exposure via breastfeeding was not associated with significant changes in bone markers; longitudinal monitoring is recommended
Kapito-Tembo et al., 2021 (38)	385 breastfed infants (260 HIV- and ARV-exposed, 125 unexposed)	Maternal TDF + EFV under Option B+	Birth to 18 months; serial assessments	No significant differences in growth, neurodevelopment, renal or bone outcomes; transient lower LAZ at 6 weeks; no fractures	Prolonged TDF/EFV exposure via breastfeeding did not adversely affect infant bone health, growth, neurodevelopment, or renal function
Kinai et al., 2021 (40)	63 mother–infant pairs (53 TDF, 10 AZT)	Maternal TDF- or AZT-based ART	Pregnancy; follow-up to 18 months	Maternal TDF linked to slightly higher infant ALP; no differences in growth, renal function, or wrist radiographs; AZT/LPV/r exposure impaired growth and bone	Maternal TDF use did not adversely affect infant bone health, growth, or renal function; AZT and LPV/r had negative effects
Vhembo et al., 2023 (34)	400 infants in PROMISE P1084s substudy	Maternal TDF-based ART (mART) vs infant nevirapine prophylaxis	Breastfeeding; follow-up to Week 74	LS-BMC at Week 26 was lower in mART group (2.64 g vs 2.77 g); no significant differences in CrCl, calcium, or phosphate	Breastfeeding exposure to TDF modestly reduced BMC but did not affect renal function; monitoring is recommended
Wang et al., 2020 (36)	128 pregnant women with high HBV viral load	TDF 300 mg/day from 28 weeks of gestation	Treatment until delivery; infants followed up to 12 months	Maternal HBV DNA decreased significantly; 50/72 mothers achieved HBV DNA negativity; no HBsAg-positive infants in treatment group; no significant differences in infant calcium, phosphorus, or BMD between breastfed and formula-fed groups	TDF effectively prevented HBV MTCT; breastfeeding during TDF therapy had no adverse impact on infant bone mineral density
Salvadori et al., 2019 (31)	137 infants born to HBV-infected mothers (Thailand)	Maternal TDF vs placebo	28 weeks of gestation to 2 months postpartum; infant lumbar spine BMD at 12 months	Infant LS BMD did not differ between TDF and placebo groups	Maternal TDF use was not associated with adverse effects on infant bone health at 12 months
Gibb et al., 2012 (25)	182 infants born to 152 HIV-infected women (Uganda/Zimbabwe)	Maternal ART with or without tenofovir	*In utero*; median 25 months follow-up	Growth (weight, height, MUAC, head circumference) normal; mild/rare renal abnormalities; no bone fractures; congenital abnormalities 3%; preterm 10%	*In utero* tenofovir exposure did not adversely affect infant skeletal development, growth, or bone health

ALP, alkaline phosphatase; ART, antiretroviral therapy; ARV, antiretroviral; AZT, zidovudine; BAP, bone alkaline phosphatase; BMD, bone mineral density; CrCl, creatinine clearance; CTX, C-terminal telopeptide of type I collagen; DXA, dual-energy X-ray absorptiometry; EFV, efavirenz; HBsAg, hepatitis B surface antigen; HBV, hepatitis B virus; LAZ, length-for-age *z*-score; LDT, telbivudine; LPV/r, lopinavir/ritonavir; LS, lumbar spine; MTCT, mother-to-child transmission; MUAC, mid-upper arm circumference; PROMISE, Promoting Maternal and Infant Survival Everywhere; TDF, tenofovir disoproxil fumarate.

### 3.5 Maternal bone health during pregnancy and postpartum

In studies examining maternal bone health during pregnancy and the postpartum period, TDF-based ART was frequently associated with accentuated bone loss and incomplete recovery. A longitudinal observational study in Ugandan women provided insight into the potential mechanism, reporting that women with HIV receiving tenofovir-based ART had higher milk calcium concentrations in early lactation and a steeper decline over the first year postpartum compared to HIV-uninfected women, reflecting increased maternal bone mineral mobilisation (44). A longitudinal study among Ugandan WWH receiving TDF-based ART initiated during pregnancy reported greater reductions in areal BMD at the total hip and whole-body-less-head during lactation compared to HIV-negative controls, with only partial recovery at three months postpartum, despite similar lumbar spine BMD changes between groups (45). Corresponding biochemical analyses revealed that WWH exhibited elevated bone resorption markers, reduced bone formation markers, and altered mineral metabolism, consistent with TDF-related effects on bone and kidney function (16).

Another long-term analysis of African breastfeeding WWH on lifelong TDF-based ART indicated that although lumbar spine BMD recovered fully and exceeded baseline levels after a median follow-up of 4.6 years, total hip and femoral neck BMD remained below early postpartum values, suggesting site-specific and persistent deficits (43). Similarly, in a randomised sub-study of the PROMISE trial, maternal TDF-ART during breastfeeding led to significantly greater declines in both lumbar spine and hip BMD by 74 weeks postpartum compared to infant nevirapine prophylaxis (39).

However, not all studies reported significant detrimental effects. A secondary analysis of the CAP016 randomised controlled trial in South African breastfeeding women found that initiating TDF/FTC as pre-exposure prophylaxis during pregnancy did not result in significant differences in lumbar spine or hip BMD or content up to 74 weeks postpartum, compared to deferred initiation, and no dose-effect relationship with tenofovir diphosphate levels was observed (41). A phase II randomised controlled trial in pregnant women with HIV-HBV co-infection found no significant differences in lumbar spine or femoral neck BMD at delivery or six months postpartum between those receiving a TDF-containing regimen and those on an AZT-based regimen, suggesting that short-term TDF exposure during pregnancy did not adversely affect maternal bone density (46). A randomised, double-blind, placebo-controlled trial conducted in Thailand among mothers with chronic HBV infection found no significant differences in maternal hip or lumbar spine BMD at 12 months postpartum between those who received TDF from 28 weeks of gestation to 2 months postpartum and the placebo group (31). In the International Maternal Paediatric Adolescent AIDS clinical trials (IMPAACT) 2010, although women receiving TDF-containing regimens showed lower hip and spine BMD at 50 weeks postpartum compared with those on tenofovir alafenamide (TAF)-based regimens, no statistically significant differences in BMD Z-scores were observed between treatment arms, and no bone fractures were reported (42). Pharmacovigilance analyses further highlighted TDF’s stronger association with bone loss compared to TAF (15).

Infant bone outcomes following *in utero* TDF exposure were also investigated. In the Virologic Efficacy and Safety of ART Combinations with TAF/TDF, EFV, and DTG (VESTED) trial, infants born to mothers receiving dolutegravir with TDF showed improved spine BMC and growth outcomes compared to those exposed to efavirenz-based TDF regimens, with no significant renal toxicity observed (7). Similarly, a Vietnamese study found no adverse effects of maternal TDF use on infant bone health, growth, or renal function, in contrast to associations observed with AZT and lopinavir/ritonavir exposure (40). Further supporting the absence of major neonatal bone toxicity, a retrospective study from Beijing among pregnant women with hepatitis B virus infection reported that TDF therapy was associated with elevated maternal ALP levels, but no significant differences in neonatal BMD were observed, regardless of the timing of TDF initiation (18).

Taken together, evidence indicated that maternal TDF-based ART during pregnancy and lactation was associated with accentuated bone loss and incomplete skeletal recovery, particularly at the hip. The observed biochemical and densitometric changes highlighted the potential for cumulative effects on maternal bone health, underscoring the need for long-term monitoring in women initiating or continuing TDF during reproductive periods. These findings are summarised in **Table 4**, which provides an overview of study designs, interventions, follow-up durations, and main outcomes related to maternal bone health during pregnancy and the postpartum period.

**Table 4:** Summary of studies evaluating maternal bone health during pregnancy and postpartum with TDF-based ART.

Study (year)	Population/model	Intervention	Exposure/duration	Key findings	Conclusion
Nabwire et al., 2020 (45)	100 Ugandan WWH on ART; 100 HIV-negative REF	Tenofovir-based ART in WWH; no ART in REF	Initiated in pregnancy; BMD assessed at 2, 14, 26 weeks of lactation and 3 months post-lactation	Both groups lost bone during lactation; WWH had greater decreases at TH and WBLH; LS reductions were similar. Post-lactation, LS recovered in both groups, but TH/WBLH recovered only in REF	TDF-ART was associated with accentuated lactational bone loss and incomplete recovery in WWH
Nabwire et al., 2023(16)	164 Ugandan women (83 WWH on TDF-ART, 81 REF)	Maternal TDF-based ART during pregnancy	Monitored through late pregnancy, lactation, ≥3 months post-lactation	WWH showed higher PTH, CTX, BALP, TALP; lower P1NP, phosphate, eGFR; altered Ca/P handling; P1NP/CTX ratio indicated increased resorption; greater BMD loss and incomplete recovery	TDF-ART was associated with increased bone resorption, impaired bone formation, and partial skeletal recovery, suggesting potential long-term risks
Matovu Kiweewa et al., 2025 (43)	519 African breastfeeding WWH	Life-long ART, predominantly TDF-based	Median follow-up 4.6 years postpartum	LS BMD fully recovered; TH and FN remained below baseline. Recovery slowed by new pregnancies and Depo-Provera; unaffected by breastfeeding duration, BMI, or cumulative TDF exposure	TDF-ART was generally safe but persistent TH/FN deficits warranted long-term bone monitoring
Stranix-Chibanda et al., 2021 (39)	398 breastfeeding WWH (PROMISE/IMPAACT P1084s sub-study)	Maternal TDF-ART vs infant nevirapine	Postpartum, weeks 1–74	Maternal TDF-ART led to greater LS BMD decline (−2.86%) and hip BMD decline (−2.29%) vs control; effects persisted after adjustment	Postpartum TDF-ART during breastfeeding was associated with greater maternal BMD loss
Masheto et al., 2024 (42)	643 pregnant WWH (9 countries, 74% breastfeeding)	Randomised to DTG + FTC/TAF, DTG + FTC/TDF, or EFV/FTC/TDF	Initiated in pregnancy; followed to 50 weeks postpartum	Hip and spine BMD Z-scores were slightly higher with TAF, lower with TDF/EFV; no significant differences across groups; renal function stable; no fractures	TDF during pregnancy and postpartum was not associated with adverse maternal bone outcomes compared with TAF or EFV
Zhu et al., 2025 (15)	643 pregnant WWH and infants across 9 countries	Maternal ART: DTG + FTC/TAF, DTG + FTC/TDF, EFV/FTC/TDF	ART from 14–28 weeks of gestation to 50 weeks postpartum; infant DXA at 26 weeks	Spine BMC, LAZ and WAZ were higher in infants exposed to DTG-based ART than EFV/FTC/TDF; fewer stunted infants; no renal differences	Maternal DTG-based ART, including TDF exposure, improved infant bone mineralisation and growth without adverse renal effects
Mbengeranwa et al., 2025 (7)	63 mother–infant pairs (53 TDF, 10 AZT)	Maternal TDF- or AZT-based ART	Pregnancy; follow-up at birth, 3, 12, and 18 months	TDF was associated with slightly higher infant ALP; no differences in growth, renal function, or wrist radiographs. AZT and LPV/r linked to impaired growth and abnormal bone findings	Maternal TDF did not adversely affect infant bone health, growth, or renal function; AZT and LPV/r negatively affected infant growth and bone
Kinai et al., 2021 (40)	FAERS post-marketing reports (2004–2023)	TDF vs TAF	Post marketing exposure; duration variable	TAF was associated with weight gain, psychiatric disorders, and maternal exposure. TDF was associated with renal disorders, foetal exposure, product issues, and bone loss; ROR for bone loss was markedly higher (714 vs 108.81 for TAF)	TDF showed a stronger pharmacovigilance signal for bone loss than TAF, underscoring the need for bone monitoring during pregnancy and long-term use
Ma et al., 2021 (18)	223 pregnant women with HBV infection (73 TDF, 150 control)	Maternal TDF therapy vs no antiviral therapy	Pregnancy, measured before delivery; neonatal BMD at birth	Maternal ALP increased with TDF; calcium and phosphorus were unchanged; neonatal BMD was unchanged; timing of TDF initiation did not affect outcomes	Maternal TDF increased ALP but did not affect neonatal bone mineral density
Salvadori et al., 2019 (31)	140 HBV-infected mothers (Thailand)	Maternal TDF vs placebo	28 weeks of gestation to 2 months postpartum; maternal hip and LS BMD at 12 months	No significant differences in maternal hip or LS BMD between groups	TDF therapy during pregnancy and postpartum did not adversely impact maternal BMD at 12 months
Wang et al., 2022 (46)	31 pregnant women with HIV/HBV co-infection in China	Maternal TDF-containing ART vs zidovudine-based ART	From 14–28 weeks of gestation to delivery; BMD measured at delivery and 6 months postpartum	No significant differences in lumbar spine or femoral neck BMD between TDF-exposed and unexposed women; adjusted analyses confirmed no effect	Short-term maternal TDF exposure during pregnancy did not adversely affect maternal bone mineral density
Nabwire et al., 2023 (44)	Ugandan lactating women, 84 WWH on TDF-based ART and 81 HIV-uninfected women	Longitudinal milk collection at 2, 14, 26, 52 weeks postpartum	Milk collection at 2, 14, 26, and 52 weeks postpartum	Women with HIV had higher milk calcium in early lactation and greater overall decline over the first year; differences in phosphorus and Ca:P ratio were smaller and mostly non-significant; milk Na and K were similar	Tenofovir-based ART in lactating women was associated with altered milk mineral composition, supporting earlier findings of increased maternal bone mineral mobilisation and potential implications for infant growth
Kistan et al., 2025 (41)	African breastfeeding women (n=300)	Maternal TDF/FTC PrEP during pregnancy	Initiated in the 2nd trimester until breastfeeding cessation	BMD and BMC at lumbar spine and hip were measured at 6, 26, 50, 74 weeks postpartum; no significant differences between the Immediate and Deferred PrEP arms; no dose-effect of TFV-DP observed	Maternal *in-utero* and postpartum exposure to TDF/FTC PrEP did not compromise bone mineral density or content

ALP, alkaline phosphatase; ART, antiretroviral therapy; AZT, zidovudine; BALP, bone-specific alkaline phosphatase; BMC, bone mineral content; BMD, bone mineral density; BMI, body mass index; Ca, calcium; CTX, C-terminal telopeptide of type I collagen; DTG, dolutegravir; DXA, dual-energy X-ray absorptiometry; EFV, efavirenz; eGFR, estimated glomerular filtration rate; FAERS, Food and Drug Administration Adverse Event Reporting System; FN, femoral neck; FTC, emtricitabine; HBV, hepatitis B virus; HIV, human immunodeficiency virus; LAZ, length-for-age z-score; LPV/r, lopinavir/ritonavir; LS, lumbar spine; P, phosphate; P1NP, procollagen type I N-terminal propeptide; PrEP, pre-exposure prophylaxis; PTH, parathyroid hormone; PROMISE/IMPAACT, Promoting Maternal and Infant Survival Everywhere/International Maternal Pediatric Adolescent AIDS Clinical Trials; REF, HIV-negative reference women; ROR, reporting odds ratio; TAF, tenofovir alafenamide; TALP, total alkaline phosphatase; TDF, tenofovir disoproxil fumarate; TH, total hip; TFV-DP, tenofovir diphosphate; WAZ, weight-for-age z-score; WBLH, whole-body-less-head; WWH, women with HIV.

## 4 Discussion

This scoping review presented the available evidence on the effects of *in utero* and lactational exposure to TDF on bone health outcomes in both offspring and mothers. The findings revealed a distinct dichotomy: for offspring, evidence was largely reassuring, indicating minimal to no lasting adverse effects on bone growth and mineralisation; in contrast, for mothers, consistent data indicated accentuated bone loss during lactation with incomplete recovery, raising concerns about long-term skeletal health.

Collectively, the evidence suggested that foetal and infant exposure to TDF, whether during pregnancy or breastfeeding, was not associated with clinically meaningful deficits in bone health in most children. Several studies demonstrated normal growth trajectories, comparable BMD in childhood, and unaltered bone metabolic markers (17, 25, 35, 37, 38), reflecting the resilience of the developing skeleton, which retained a strong capacity for growth and remodelling. The notable exception was a study reporting a 12% reduction in neonatal BMC (33), implying that TDF might exert a direct yet limited effect on foetal bone accretion. However, the absence of this finding in long-term follow-up studies suggested possible “catch-up” mineralisation during early childhood (32, 37). The clinical significance of such transient reductions in neonatal BMC remained uncertain and warranted longitudinal investigation to determine whether they affected peak bone mass attainment or fracture risk later in life. Moreover, the absence of a concentration-dependent effect, as shown by meconium tenofovir levels (26), provided additional reassurance that toxic exposure thresholds for the foetal skeleton were unlikely within clinical dosing ranges.

In contrast, findings regarding maternal bone health were more consistent and concerning. Evidence from DXA assessments (39, 43, 45) and bone turnover biomarkers (Nabwire et al., 2023) converged to show that TDF use during the high turnover state of lactation exacerbated physiological bone loss, particularly at weight-bearing sites such as the hip. The incomplete recovery of hip BMD years after weaning (43, 45) was particularly notable, as lactational bone loss is typically reversible. This pattern suggested that TDF may have altered bone remodelling dynamics in a way that increased long-term risks of osteopenia and osteoporosis among women requiring lifelong ART. Notably, initiation of TDF-based therapy during the postpartum period appeared to exert a more pronounced adverse effect on BMD than antenatal initiation (39), identifying the postpartum phase as a period of heightened vulnerability. These findings should inform clinical decision-making and highlight the importance of monitoring maternal bone health during and after lactation (48, 49).

Comparative data provided additional context for TDF’s safety profile in pregnancy. TDF appeared safer for infant bone health than AZT (40), which is relevant when selecting antiretroviral regimens during gestation. Nevertheless, pharmacovigilance data consistently indicated that TAF possessed a more favourable bone safety profile (15), likely due to its targeted intracellular delivery and reduced systemic exposure. The improved infant bone outcomes observed with TAF-based regimens in the VESTED trial (7), albeit partly confounded by efavirenz in the comparator arm, this profile aligns with it. These observations suggested that TAF might represent a preferable alternative for women at risk of bone loss, although issues of cost, accessibility, and potential metabolic effects also required consideration.

Overall, the evidence demonstrated that the impact of TDF exposure on bone health diverged between mothers and offspring. This pattern was consistent with broader observations in HIV-exposed uninfected children, who face multisystem risks, including reduced bone density (50). For infants and children, most studies found no clinically significant impairment in bone mineralisation, growth, or development following TDF exposure during gestation and breastfeeding. Occasional biochemical or densitometric changes were transient and not associated with fractures or long-term growth deficits, supporting the overall bone safety of TDF for paediatric populations in the context of HIV and HBV prevention (51).

Conversely, in mothers, TDF-based therapy during pregnancy and the postpartum period was consistently linked to greater BMD loss, particularly at the hip, with incomplete recovery after weaning. Biochemical findings and alterations in breast milk mineral composition suggested that increased bone resorption and mineral mobilisation underlay these effects. Mechanistically, TDF may have disrupted calcium homeostasis through interference with vitamin D and PTH regulation (52). Although these densitometric changes were not consistently associated with fracture risk, their potential cumulative impact warranted attention, especially as bone loss was more pronounced in women receiving TDF for HIV treatment than in those using it as PrEP. Concurrent use of depot medroxyprogesterone acetate contraception, which independently reduces BMD, may have further amplified this risk (53).

Women’s vulnerability to TDF-related bone effects likely extends beyond the reproductive period. Young adults with chronic HIV infection and long-term TDF exposure exhibited subclinical renal dysfunction and altered bone metabolism (54), suggesting possible cumulative effects over time. This was of particular concern for women experiencing repeated TDF exposure during critical stages of bone turnover, such as pregnancy, lactation, and menopause (49).

It is important to note that the included studies varied considerably in their adjustment for potential confounders. While many studies adjusted for maternal age, body mass index, and parity, adjustment for vitamin D status, calcium supplementation, concomitant medications affecting bone metabolism [e.g., corticosteroids (55), anticonvulsants (56)], and childhood nutritional status was inconsistent across studies (57). This variability in covariate adjustment represents an important source of heterogeneity and should be considered when interpreting the findings. Residual confounding remains a concern across the included studies. Vitamin D deficiency, which is prevalent in many populations studied and independently affects bone metabolism, was not consistently measured or adjusted for (57, 58). Similarly, concomitant use of medications affecting calcium metabolism, such as corticosteroids or anticonvulsants, was rarely reported (55, 59). Nutritional status in early childhood, which profoundly influences bone accrual, was variably assessed using different anthropometric metrics (60). The potential interaction between TDF exposure and these confounders could either amplify or attenuate the observed bone effects (57).

This divergence underscored that TDF exposure entailed distinct risk-benefit considerations for mothers and children. The established efficacy of TDF in preventing mother-to-child transmission of HIV and HBV, coupled with reassuring infant bone safety, supported its continued use in pregnancy and lactation. However, the potential for cumulative, site-specific bone loss in women justified a personalised approach to care, including the consideration of alternative agents such as TAF where appropriate, and long-term bone health surveillance, particularly for women with additional risk factors for osteoporosis. Future studies should clarify the long-term fracture risk and develop strategies to optimise skeletal health in women requiring TDF during their reproductive years, with an emphasis on sex-specific and vulnerable populations.

Substantial heterogeneity was observed across the included studies, arising from multiple sources. First, study designs varied considerably, including randomised controlled trials (39, 42), prospective and retrospective cohort studies (38, 45), and secondary analyses (34), each with differing levels of bias control. Second, assessment methods for bone outcomes were heterogeneous: BMD and BMC were measured using different DXA systems and skeletal sites, such as the lumbar spine and total hip (37) or whole body (33), while some studies used quantitative ultrasound (35) or biochemical markers including BAP, CTX, and PTH as surrogate endpoints (14, 35). Third, exposure definitions varied, with some studies examining TDF as part of combination ART (24) (24), others as PrEP (19), with differing durations and timing of exposure [antenatal only: (33); antenatal plus breastfeeding: (38))]. Fourth, comparison groups differed across studies, including non-TDF ART (40), no ART or placebo (31, 36), and HIV-negative controls (45), complicating direct comparisons. Fifth, population characteristics, including HIV status (27), HBV status (17), geographic region spanning Africa (38, 45) and Asia (37) as well as nutritional status and baseline bone health, varied substantially. These sources of heterogeneity precluded quantitative meta-analysis and necessitated a narrative synthesis.

Beyond areal BMD, accumulating evidence indicates that bone quality is impaired by TDF at multiple hierarchical levels, including trabecular microarchitecture and cortical geometry (61). People living with HIV on TDF treatment exhibited consistent deterioration of cortical and trabecular bone determined by HR-pQCT, trabecular bone score by DXA software and bone material strength index (BMSi) by microindentation device (62–65). A study showed that switching from TDF to TAF improved the BMSi in HIV patients after 24 weeks. This improvement might be associated with the suppression of high bone remodelling (Jade 8). However, long-term studies on the effects of *in utero* and lactational TDF exposure on bone health remain limited. Chronic liver disease complicates bone assessment by disrupting vitamin D metabolism (66) and altering signaling of liver-derived factors such as IGF-1 and TNF-α (67); the concept of hepatic osteodystrophy has been revisited as a metabolically associated bone disease induced by chronic liver disease (68). The critical yet underexplored roles of gender and sex in HIV-associated bone health are underscored by findings that women with HIV in resource-limited settings experience accelerated bone loss following initiation of TDF-containing ART during pregnancy, lactation, or menopause, while impaired bone accrual during puberty is observed in children and adolescents (49). Limited attention to bone outcomes has been reported in recent trials of TAF or integrase inhibitors, and sex-specific data on inflammation, immune activation, ART response, and bone turnover remain scarce, leaving substantial knowledge gaps regarding differential fracture risks and BMD trajectories between sexes (39, 49, 69). Therefore, sex-disaggregated analyses are recommended in future investigations to address the long-term consequences of ART exposure and low bone mass on fracture risk in the aging and increasingly diverse HIV population (48, 49).

Several limitations within the evidence base were noted. Long-term data extending into adolescence and adulthood for TDF-exposed offspring were lacking, leaving uncertainties about effects on peak bone mass and lifetime fracture risk. Much of the literature was observational, with inherent confounding despite statistical adjustments, while randomised controlled trials remained limited. Variability in assessment methods, including DXA, biomarkers, and ultrasound, complicated comparisons across studies. Furthermore, the predominance of research conducted in resource-limited settings raised questions about generalisability to populations with differing nutritional, genetic, and healthcare profiles.

This review has several limitations that should be considered when interpreting the findings. The evidence base was restricted to studies published in Mandarin and English, which may have resulted in the omission of relevant research in other languages and introduced language-related selection bias. The inclusion of CNKI may have introduced a regional focus on Chinese populations; while this captured important studies from a high-burden region, it may limit the generalisability of findings to other geographic and ethnic groups. Publication bias is also possible, as studies reporting statistically significant or positive findings are more likely to be disseminated, potentially leading to an overestimation of effect sizes. Furthermore, most included studies employed observational designs; although many applied multivariable adjustments, residual confounding cannot be fully excluded. Several important covariates that may influence bone health in TDF-exposed populations were inconsistently reported or not adjusted for across the included studies. Chronic comorbidities such as liver disease (particularly in HBV-infected populations), metabolic syndrome, obesity, and viral coinfections (e.g., HIV-HBV or HIV-HCV coinfection) are known to independently affect bone metabolism and may confound the observed associations between TDF exposure and bone outcomes. Lifestyle factors, including smoking, alcohol consumption, and physical activity levels, which are established risk factors for bone loss, were also variably reported. The potential interaction between TDF-related and infections remains, and these covariates are incompletely characterised, limiting the ability to isolate the direct skeletal impact of TDF from these confounding influences. Randomised controlled trials examining the effects of TDF exposure on bone health remain scarce, and the limited use of double-blind designs restricts the strength of causal inference. Long-term data on individuals exposed to TDF *in utero* or during breastfeeding are insufficient, particularly regarding follow-up into adolescence and adulthood, leaving the implications for peak bone mass and lifelong fracture risk unresolved. Methodological heterogeneity across studies, including differences in DXA systems, bone turnover markers, and ultrasonographic assessments, further complicates direct comparison and limits the feasibility of meta-analysis. Finally, as most studies were conducted in resource-limited settings, the generalisability of the findings to populations with different nutritional profiles, genetic backgrounds, and healthcare infrastructures should be approached with caution. Formal risk of bias assessment was not performed, consistent with the scoping review methodology, which prioritises evidence mapping over critical appraisal. Consequently, the internal validity of the included studies was not systematically evaluated, and the potential for study-level bias should be considered when interpreting the findings.

The clinical implications were twofold. For offspring, findings were largely reassuring; the benefits of TDF in preventing mother-to-child transmission clearly outweighed minimal and likely transient risks to bone health. For mothers, however, heightened vigilance was warranted. Routine maternal bone health monitoring should be incorporated into HIV care, including baseline and follow-up DXA scans where feasible, alongside adequate calcium and vitamin D intake.

## 5 Conclusion

The collective evidence on the impact of *in utero* and lactational exposure to TDF on bone health presented a largely reassuring, albeit nuanced, picture. Most studies, conducted across diverse populations and settings, indicated that TDF exposure was not associated with clinically significant impairments in growth, bone mineralisation, or bone metabolism in infants and young children. Numerous observational studies and clinical trials found no substantial differences in anthropometric measures, BMC or BMD, quantitative ultrasound results, or biochemical bone markers between TDF-exposed and unexposed children, with follow-up in some cases extending to six or seven years of age. Furthermore, the evidence suggested that breastfeeding during maternal TDF therapy did not exert a significant adverse effect on infant bone outcomes. However, a small number of well-conducted studies reported subtle yet statistically significant reductions in neonatal BMC and modestly impaired bone mineral accrual in infants exposed through breastfeeding, although the clinical relevance of these findings was considered limited. For mothers, the evidence was more consistent: TDF-based antiretroviral therapy during pregnancy and lactation was associated with accentuated bone loss, particularly at the hip, and incomplete skeletal recovery postpartum compared with HIV negative women or those receiving non-TDF regimens. In summary, while TDF exposure during the perinatal period appeared to have minimal impact on paediatric bone development, it was associated with discernible effects on maternal bone metabolism, characterised by enhanced bone resorption during lactation and incomplete recovery thereafter. This highlighted the importance of monitoring maternal bone health during TDF use. Overall, the benefits of TDF in preventing mother-to-child transmission of HIV and HBV outweighed the potential bone-related risks, which, for the child, appeared transient and of limited clinical significance in most cases, though long-term paediatric follow-up studies remained warranted.
